# Exploring natural sunscreens: UVB protection and antioxidant properties in gadusol‐rich fish roes extracts

**DOI:** 10.1111/php.70042

**Published:** 2025-10-06

**Authors:** Rocío Isla Naveira, Gabriela Hollmann, José María Monserrat, Ana Paula S. Votto, Julie Medeiros da Silveira, Andressa Mai Matsumoto, Lais Zortéa, Andy Joel Taipe Huisa, Agueda E. Massa, M. Sandra Churio

**Affiliations:** ^1^ Instituto Nacional de Investigación y Desarrollo Pesquero (INIDEP) Mar del Plata Argentina; ^2^ Instituto de Investigaciones Marinas y Costeras (IIMyC), UNMDP – CONICET Mar del Plata Argentina; ^3^ Instituto de Ciências Biológicas (ICB), Universidade Federal Do Rio Grande – FURG Rio Grande Brazil; ^4^ Programa de Pós‐graduação Em Ciências Fisiológicas Universidade Federal Do Rio Grande – FURG Rio Grande Brazil; ^5^ Departamento de Química y Bioquímica, FCEyN UNMDP Mar del Plata Argentina; ^6^ Instituto de Investigaciones Físicas de Mar del Plata (IFIMAR), UNMDP – CONICET Mar del Plata Argentina

**Keywords:** antioxidant, bioactive molecules, by‐products, *Caenorhabditis elegans*, HaCat cell line, in vivo assays, UV‐photoprotector

## Abstract

The search for natural alternatives to synthetic sunscreens has driven interest in marine compounds with antioxidant and UV‐protective properties. The present study expands our understanding of the potential of gadusol by evaluating the photochemoprotective and antioxidant effects of extracts from an underexploited marine by‐product: the roes of yellowtail amberjack fish (*Seriola lalandi*). UVB‐mediated responses were studied in vitro and in vivo using HaCaT keratinocytes and *Caenorhabditis elegans* worms, respectively. Additionally, several antioxidant assays were conducted to evaluate the antioxidant capacity and thermal stability over time. We also tested the docking binding of gadusol to the Nrf2‐binding domain of Keap1 to better understand its potential chemoprotective role. Overall, the gadusol‐containing extracts exhibited remarkable stability over time, offering effective protection against UVB radiation in both in vitro and in vivo models. This information contributes to a better characterization of the functional role of gadusol in crude extracts and its relevance for the design of innovative applications in various pharmaceutical and cosmetic industries.

## Graphical abstract

Gadusol‐rich extracts provide effective UVB protection and antioxidant activity in HaCaT cells and *C. elegans*, highlighting their potential as natural photochemoprotective agents for pharmaceutical and cosmetic applications. It also demonstrates remarkable in vitro antioxidant capacity and thermal stability over time.

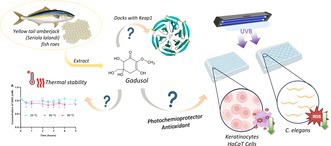

**Abbreviations**
ABTS: 2,2′‐azino‐bis(3‐ethylbenzothiazoline‐6‐sulfonic acid)
ARE: antioxidant response element
DGR: Double Glycine Repeat
ET: electron transfer reactions
GAE: gallic acid equivalent antioxidant capacity
LGA: Lamarckian genetic algorithm
MAAs: mycosporine‐like amino acids
MTT: 3‐(4,5‐dimethylthiazolyl‐2)‐2,5‐diphenyltetrazolium bromide
NGM: nematode growth médium
Nrf2: Transcriptional NF‐E2‐related factor‐2
ROS: reactive oxygen species
SPF: Sun Protection Factor
TBF: tartary buckwheat flavone
TEAC: 6‐hydroxy‐2,5,7,8‐tetramethylchroman‐2‐carboxylic acid equivalent antioxidant capacity
TROLOX: 6‐hydroxy‐2,5,7,8‐tetramethylchroman‐2‐carboxylic acid
UV: ultraviolet

## INTRODUCTION

Exposure to ultraviolet (UV) radiation generates reactive oxygen species (ROS) within cells, which are detrimental to cellular integrity, leading to mutations and alterations in gene expression. Oxidative stress can disrupt essential metabolic processes such as protein synthesis and energy production. These metabolic disruptions can impair cell function, contribute to premature aging, and increase the risk of skin cancer.^1^ Modern sunscreens provide broad‐spectrum UV protection in the UVB (280–320 nm) and UVA (320–400 nm) regions and are widely used in pharmaceutical and cosmetic personal care products as well as in the food industry for product packaging, among other applications.^2^

However, in recent decades, contamination of aquatic environments by some of these photoprotective substances has increased dramatically, becoming catalogued as persistent pollutants. Over the past few years, numerous studies have been conducted regarding the adverse effects of different synthetic sunscreens and the different problems that affect marine and terrestrial environments, including genotoxic effects, endocrine disruption, and photoallergy.^3^, ^4^, ^5^ Furthermore, the use of natural products as substitutes for synthetic ones is increasing, and consumers are becoming more aware of their benefits. Regarding ecological standards, reducing pollution, materials, and energy consumption aligns with the circular economy model and the 2030 agenda for sustainable development.^6^, ^7^ In this sense, the growth of aquaculture worldwide also needs to assess the utilization of by‐products and the potential of distinct species, such as *Seriola* spp., which have a history of being successfully farmed in various countries, creating an opportunity.^8^, ^9^

As natural photoprotectants, a particular group of compounds has attracted attention lately: gadusol and its deprotonated anionic form, gadusolate.^10^, ^11^ They are secondary metabolites of marine organisms with intense UVB absorption and exhibit biosynthetic and structural similarities to mycosporine‐like amino acids (MAAs).^12^ Additionally, the capacity of gadusol to quench singlet oxygen, a key ROS that initiates oxidative reactions and generates other ROS, has been well established in aqueous solutions.^13^ Several studies support the high photostability of these metabolites and their rapid deactivation through the efficient dissipation of light energy as heat.^10^, ^14^ This is a critical requirement for the development of efficient UVR filters. Moreover, the deprotonated anionic form, gadusolate, is the major species under physiological pH conditions, and it is the most appropriate for boosting the Sun Protection Factor (SPF) value.^3^ Building on its distinctive physicochemical properties, gadusol's natural origin and functional role in vivo provide further insight into its biological significance. Gadusol is found in high concentrations in fish roes from various marine species^15^, ^16^ and stands out among other antioxidant compounds, such as peptides and enzymes.

Nearly a decade ago, the genes responsible for gadusol synthesis in fish and other vertebrates were identified.^17^ Nevertheless, literature regarding the stability and bioactivity of gadusol in organisms and cellular lines is scarce. Recently, it was revealed that gadusol plays an important functional role in embryonic development, based on its UV‐photoprotective and antioxidant abilities, to guarantee the survival of fish embryos.^18^ In addition, Shaw et al.^19^ investigated the ability of shinorine, a related MAA, to disrupt Nrf2‐Keap1 interaction in zebrafish hepatocytes. Transcriptional NF‐E2‐related factor‐2 (Nrf2) binds to the antioxidant response element (ARE) in the promoter region of genes encoding antioxidant enzymes.^20^ Given that ROS production can activate the Nrf2‐Keap1 pathway, this system plays a crucial role in defense against oxidative stress and inflammation.

In this context, we designed the study to assess the photochemoprotective and antioxidant effects of gadusol extracts by elucidating their interactions with biological targets at molecular, cellular, and organismal levels. We evaluated UVB‐induced damage in HaCaT keratinocytes and confirmed photoprotective efficacy through in vivo assays in *Caenorhabditis elegans*. The first model is a human keratinocyte cell line that has been widely used to study skin biology and inflammatory responses.^21^ *C. elegans* is a soil‐dwelling, free‐living worm extensively utilized to assess photoaging damage.^22^ Correspondingly, to gain insight into the mechanisms underlying cellular responses, we analyzed the docking affinity of gadusolate for Keap1, considering that ROS production can activate the Nrf2‐Keap1 pathway.^23^ This approach aimed to clarify the chemoprotective potential of gadusol by examining its capacity to modulate this pathway under UVB stress. In addition, we analyzed the stability of the extracts through in vitro antioxidant assays over time and under different conditions to determine the optimal storage methods for potential applications of gadusol‐containing preparations. Consequently, this study not only advances our understanding of the chemistry and bioactivity of natural sunscreens but also provides practical guidance for developing innovative gadusol‐based products in the pharmaceutical, cosmetic, and food industries.

## MATERIALS AND METHODS

### Sampling and extraction

Fish roes from spawning yellowtail amberjack (*Seriola lalandi*) were collected from an aquaculture station at the “Instituto Nacional de Investigación y Desarrollo Pesquero” (INIDEP) in Mar del Plata. Collections were made from four different spawning events between January and February 2023.

The crude extracts were obtained, following the protocol of Plack et al.^24^ with modifications, by homogenizing the tissue in a proportion of 0.25 g mL^−1^ of 100% ethanol (Sintorgan®) for 3 min in an Omni mixer (OMNI International®) and sonicating (Ultrasonic Cole‐Parmer® 8892) for 10 min, then left for 48 h at 4°C. The homogenate was filtered, and the supernatant was concentrated in a rotary evaporator to dryness at reduced pressure in a bath at 40°C. Subsequently, it was resuspended in 80% ethanol and filtered using an SPE Welchrom C18E cartridge. Finally, the extract was concentrated again in a rotary evaporator under the same conditions as before and freeze‐dried to obtain a yellowish powder of the crude extract with gadusol.

### Instrumental analysis

Preliminary identification and quantification of gadusol were performed using UV–visible spectroscopy (Shimadzu® UV‐1800), verifying the absorbance maximum at 296 nm at pH 8.2 with a characteristic reversible shift to 268 nm upon decreasing the pH to 2.5. The concentration of gadusol in the extracts was estimated based on the absorbance at 268 nm and pH 2.5, and using a molar absorption coefficient of 12,400 M^−1^ cm^−1^.^24^

Also, reverse‐phase high‐performance liquid chromatography was performed (HPLC, Shimadzu® CBM‐20A, LC‐20AT pump, SIL‐10AF auto‐sampler) with a VP‐ODS Shim‐pack Shimadzu® C18 column (150 mm × 4.6 mm i.d. × 5 μm) protected by a guard column with a GVP‐ODS Shim‐pack Shimadzu® C18 cartridge (4.6 mm i.d. × 10 mm), both thermostated at 15°C (Shimadzu® CTO‐10AS‐VP oven). The mobile phase was an aqueous solution of 0.35 M acetic acid (Sintorgan®), pH 2.5 with an isocratic flow of 1.4 mL min^−1^ for 10 min. Detection was performed at 268 nm using a UV–visible detector (Shimadzu SPD‐20A) and contrasted with a self‐prepared gadusol standard.^15^

### In vitro antioxidant activity

Three assays were performed to study the antioxidant activity of the crude extracts in vitro. The Folin–Ciocalteu reduction assay, described by Huang et al. (2005),^25^ and the ferrous ion‐chelating capacity assay reported by Min et al. (2011),^26^ were carried out to determine the gallic acid equivalent antioxidant capacity (GAE). In addition, the 6‐hydroxy‐2,5,7,8‐tetramethylchroman‐2‐carboxylic acid (TROLOX) equivalent antioxidant capacity (TEAC) was evaluated by measuring the absorbance of the synthetic oxidant agent ABTS^•+^ (the cation radical of 2,2′‐azino‐bis(3‐ethylbenzothiazoline‐6‐sulfonic acid)).^27^ All three assays were performed in triplicate as previously reported.^28^

### In vivo activity evaluated in *C. elegans*

The in vivo activity of crude extracts was assessed using *C. elegans* worms N2, Bristol (wild strain), as a model animal. The nematodes were cultured in Petri dishes containing nematode growth medium (NGM; 3.0 g L^−1^ NaCl, 5.0 g L^−1^ peptone, 0.005 g L^−1^ cholesterol, dissolved in absolute ethanol, 0.11 g L^−1^ CaCl_2_, 0.12 g L^−1^ MgSO_4_, 5.3 g L^−1^ KH_2_PO_4_, 17.0 g L^−1^ agar, pH 6.0, with 1 mL of antibiotic‐antimycotic solution) and kept at controlled temperature in an incubator at 20°C. The plates were seeded with *Escherichia coli* bacteria, non‐pathogenic strain OP50, with an optical density of 1.0 at 600 nm, as a food source for *C. elegans*, according to the traditional maintenance procedures described by Brenner.^29^ To ensure that all organisms were at the same developmental stage for UVB exposure, they were filtered through a 5 μm mesh, allowing only those in the larval (L1) stage to pass through.^30^ Following filtration, the ISO (2020) method^31^ was used to estimate the number of organisms needed for exposure, with the aim of 60 per replicate, carried out in triplicates. The experiments were performed in 24‐well plates containing NGM solid medium and M9 buffer (3 g L^−1^ KH_2_PO_4_, 6 g L^−1^ Na_2_HPO_4_, 5 g L^−1^ NaCl, 1 mM MgSO_4_), and the estimated number of organisms was placed in each well.^31^

Furthermore, the freeze‐dried extract was resuspended in M9 buffer to obtain a final concentration of 1 mM of gadusol for *C. elegans* exposure in each well of the plate. A control plate containing only M9 buffer was also prepared. The irradiation system consisted of a UVB lamp (VL 115 L: 115 V, 30 W Vilber Lourmat, France; λ = 280–320 nm, peak 312 nm). The UV dose was measured using a radiometer (ILT2400, International Light Technologies). Two doses of UVB radiation were tested: D_0_ = 0.06 J cm^−2^ and D_1_ = 0.36 J cm^−2^, with a control kept in darkness.

After exposure, the worms were incubated at 20°C for 24 h, washed from the plates with M9 buffer, and centrifuged. Mortality was determined using SYBR® green dye under a fluorescence microscope (Olympus IX2‐UCB/U‐HSTR2).^32^ The concentration of ROS was measured using 2,7‐dichlorofluoresceindiacetate (H2DCF‐DA; Sigma‐Aldrich) and analyzed with fluorescence microscopy^33^ as well as the concentration of lipofuscin. The fluorescence intensities were evaluated using open‐source Image J software.

### In vitro keratinocytes HaCat cell line testing

The in vitro cellular effects of the extract were explored in the human keratinocytes HaCaT cell line. The cells were cultured at 37°C and 5% CO_2_ in disposable plastic flasks filled with DMEM medium, supplemented with 10% fetal bovine serum and 1% antibiotic‐antimycotic (penicillin, streptomycin, and amphotericin), at the Federal University of Rio Grande's Cell Culture Laboratory.

The cells were seeded into 96‐well culture plates (5 × 10^4^ cells mL^−1^) and allowed to grow for 24 h in 200 μL of medium. After that time, the cells were washed with PBS buffer of 0.2 g L^−1^ KH_2_PO_4_, 1.15 g L^−1^ Na_2_HPO_5_.7.H_2_O, 8 g L^−1^ NaCl, 0.2 g L^−1^KCl. Prior to irradiation, the cells were covered with 190 μL of PBS and 10 μL of extracts containing different concentrations of gadusol, to obtain final concentrations of C_1_ = 0.01 mM, C_2_ = 0.05 mM, and C_3_ = 0.10 mM. The control group received 10 μL of sterile water. The irradiation system consisted of the same UVB lamp and radiometer used in the *C. elegans* experiments. Two doses were analyzed for the study, D_1_ = 0.06 J cm^−2^ and D_2_ = 0.12 J cm^−2^. In parallel, non‐irradiated control cells were treated similarly, but the wells were covered with dark protection to prevent irradiation. After irradiation, cells were washed with PBS and allowed to grow in 200 μL of DMEM medium containing the same concentration of gadusol as before for 24 h. Complementary experiments comparing the conditions without the presence of gadusol during the 24 h after irradiation were conducted.

For each assay, cell viability was determined using the MTT (3‐(4,5‐dimethylthiazolyl‐2)‐2,5‐diphenyltetrazolium bromide) assay. Briefly, after discarding the medium, 180 μL of fresh medium and 20 μL of MTT (5 mg mL^−1^ PBS) were added to each well and incubated for 3 h. The medium was then removed and 200 μL of DMSO was added to solubilize the formazan product. Finally, absorbance was measured at 490 nm using a microplate reader (Biotek ELx 800).

### Molecular docking study

The chemical structure of gadusolate was obtained from ZINC (ZINC100829654) and PubChem (PubChem CID: 85926716) databases. The ligand was prepared using Avogadro. The structures of both Keap1a and Keap1b of zebrafish were obtained from the AlphaFold Protein Structure Database (Keap1A: AF‐Q1ECZ2‐F1‐v4 and Keap1B: AF‐A0A2R8Q1W5‐F1‐v4), and both proteins were prepared using AutoDockTools −1.5.7. To perform the docking, AutoDock 4.2 software was used. The grid box was designed to cover the amino acid residues of the DGR domain, which are crucial for the interaction with the ETGE and DLG motifs of Nrf2 for Keap1a and Keap1b as described by Shaw et al^19^ The dimensions of the x, y, and z‐axis grids were 70 × 70 × 70 Å, and the distance between the two connecting grid points was 0.375 Å. The Lamarckian genetic algorithm (LGA) was used in AutoDock4 for receptor‐fixed ligand‐flexible docking calculations. For each protein‐ligand docking, a maximum of 50 binding poses were generated, and the most suitable pose was chosen based on the binding energy interaction between the active site amino acid residues and ligands. Discovery Studio Visualizer was used for the visualization and analysis of the docked protein‐ligand complex.

### Stability analysis of the extracts

The storage effect and thermal stability of the gadusol extracts were assessed. The stability of the crude extracts at a concentration of 1 mM of gadusol at two pH values (2.5 and 6.8) was evaluated at 4°C, −21°C, and −80°C for 6 months by monitoring gadusol concentration (UV–Vis, Shimadzu® UV‐1800) and antioxidant capacity (GAE and TEAC) as previously described. Thermal stability was determined by quantifying gadusol in extracts maintained at 25, 60, and 90°C in a thermostatic bath with an air atmosphere for 4 h. Additionally, a 7‐day analysis in a dry bath at the intermediate temperature (60°C) was performed.

### Statistical analysis

Statistical analysis of the results of *C. elegans* and the cell line was performed with RStudio software^34^ by means of ANOVA and Tukey's test after verification of normality and homogeneity of variance. When required, the data were transformed to verify assumptions. Assumptions of normality and homogeneity of variance were verified for every variable using the Shapiro‐Wilks test and residual plots analysis, respectively.

The results of the analysis of stability over time and temperature were performed using two‐way mixed ANOVA (fixed factors: pH and temperature, random factor: time), with post hoc Tukey test (α = 0.05).

## RESULTS

### Gadusol content and in vitro antioxidant activity of the extract

**FIGURE 1:**
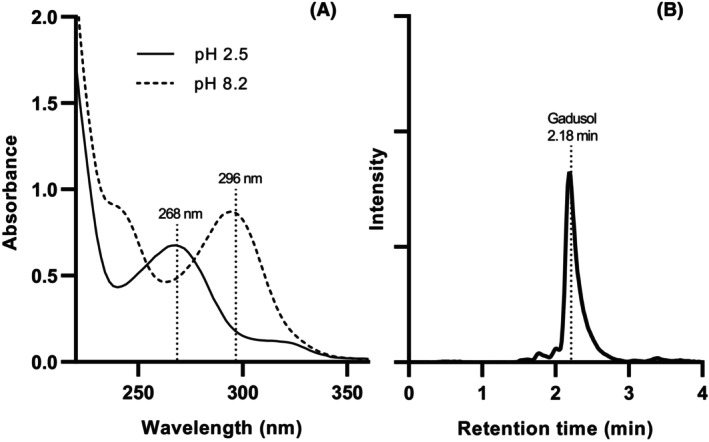
(A) Absorption spectra at pH 2.5 (gadusol) and pH 8.2 (gadusolate). (B) HPLC‐UV–vis (detection at 268 nm). Gadusol eluted at a 2.18 min retention time.

Gadusol was obtained with a yield of 4.56 ± 0.16 mg gadusol g^−1^ dry weight of tissue. Identification and quantification were achieved through UV–visible spectra, by verifying both the characteristic solvatochromic shift with a maximum at 268 nm for gadusol at pH 2.5 and a maximum of 296 nm for gadusolate at pH 8.2 (Figure 1A) and a retention time of 2.18 min in the chromatogram (Figure 1B).

The results of the antioxidant properties of extracts containing gadusol in three in vitro assays are shown in Table 1.

**TABLE 1:** Antioxidant activity of gadusol extracts evaluated through different assays (ABTS) over time, Folin–Ciocalteu and ferrous ion‐chelating capacity. Values are expressed as mM TROLOX/mM gadusol (TEAC, trolox equivalent antioxidant capacity) or mM galic acid/mM gadusol (GAE, gallic acid equivalent antioxidant capacity), (mean ± SD, *n* = 3).

**ABTS–TEAC values**		
10 min	30 min	60 min
0.60 ± 0.07	0.74 ± 0.07	0.86 ± 0.08
**Folin–Ciocalteu – GAE value**		
1.01 ± 0.05		
**Ferrous ion‐chelating capacity – GAE values**		
0.48 ± 0.04		

### In vivo photoprotective activity

**FIGURE 2:**
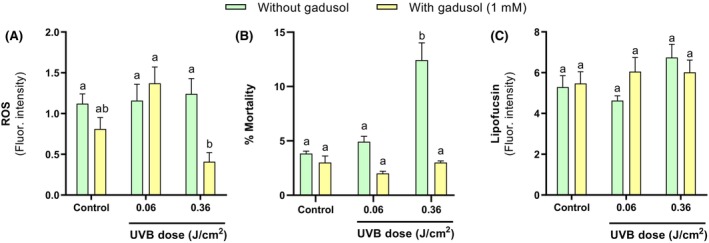
(A) Quantifications of reactive oxygen species (ROS) by fluorescent intensity. (B) Percentage of mortality in *Caenorhabditis elegans*. (C) Lipofuscin quantified as fluorescent intensity. Different superscript letters (a and b) indicate significant differences between treatments (*p* < 0.05).

The photoprotective effects of gadusol on the physiology of worms exposed to UVB were assessed to determine the role of oxidative stress. The results of the three assays are shown in Figure 2. The analysis revealed significant differences in ROS production for animals exposed to higher UVB doses (0.36 J cm^−2^), compared to those in the presence of 1 mM gadusol (Figure 2A). It also showed an effect on the mortality test, at the higher doses of UVB, with significant differences (*p* < 0.05) between the control group (without gadusol) and treatment with 1 mM of gadusol extract, with a reduction of 10% in mortality (Figure 2B). In contrast, as shown in Figure 2C, there were no significant differences in lipofuscin content among the different treatments, including the control group.

### In vitro gadusol activity evaluated in keratocytes HaCat and molecular docking

**FIGURE 3:**
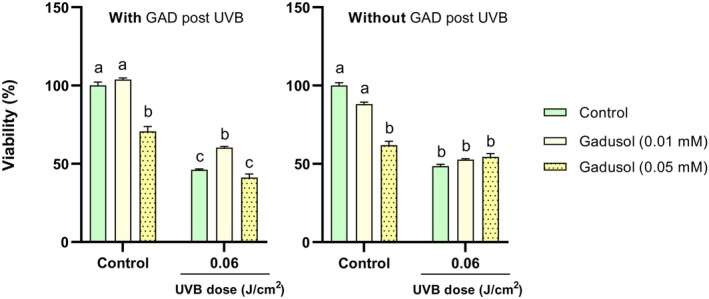
Percentage of viability of HaCaT cells 24 h after irradiation (post UVB) with the lowest dose of UVB with or without gadusol (GAD) during the 24 h after irradiation. Different superscript letters (a, b, and c) indicate significant differences (*p* < 0.05).

The in vitro cellular effects of the extract are presented in Figure 3 which shows the viability of HaCaT cells 24 h after irradiation (0.06 J cm^−2^ UVB dose) with or without gadusol after irradiation time. The results show that gadusol, at a concentration of 0.01 mM, does not cause cytotoxicity and significantly protects cells against damage when used during and after irradiation (Figure 3, left). The concentration of 0.05 mM reduced cell viability by 25%, revealing a cytotoxic effect, and the effect at 0.05 mM gadusol was not greater than that of the irradiated control. In addition, no significant differences were observed when gadusol was present through irradiation but not during the 24 h after irradiation (Figure 3, right). Comparison between the two doses of UVB and gadusol after irradiation was also achieved (see Figure S1). The highest dose of UVB showed a powerful effect on the cells, with a reduction of more than 50% in viability for the control and the two gadusol concentrations tested. In addition, the concentration of 0.10 mM of gadusol promoted cytotoxicity, with a reduction of 80% in cell viability (Figure S1).

Our docking results showed no interaction between the ligand and amino acid residues of the DGR domain of Keap1. However, 10 H‐bonds interacted with Ala340, Val391, Val438, Val485, Val532, Val579, and gadusolate (the major species under physiological pH conditions), with a score of −6.88 kcal/mol for Keap1a. Also, the Keap1b showed six H‐bond interactions, but with different residues, like Ala332, Val383, Val430, Val524, Val571, with a score of −6.58 kcal/mol (Figure S2).

### Stability over time analysis of the extracts

**FIGURE 4:**
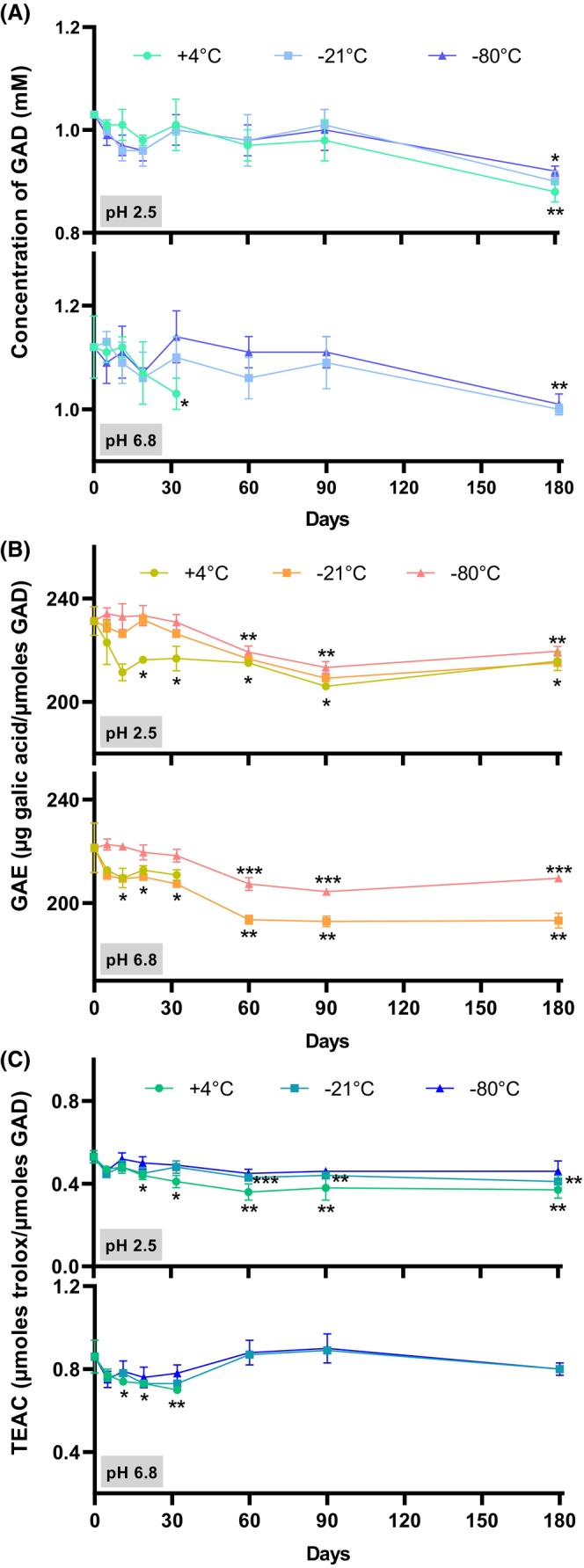
Temperature effect (+4°C, −21°C, and −80°C) on the stability and activity of the crude extract over storage time (180 days) at pH 2.5 and 6.8. (A) Concentration of gadusol (mM); (B) Antioxidant activity as μM gallic acid/μM gadusol (GAE). (C) Antioxidant activity as mM Trolox/mM gadusol (TEAC) over storage time at three temperatures (+4°C, −21°C, and −80°C). Asterisks indicate statistical differences over time at the same temperature. Different letters indicate significant differences between temperatures (two‐way mixed ANOVA with post hoc Tukey test, *p* < 0.05).

The stability of gadusol species in the extracts and their antioxidant activity under various storage conditions over 180 days is shown in Figure 4. At storage temperatures of +4°C and −80°C, and pH 6.8, significant differences in gadusol concentration were observed after 30 days, with a lower decrease at −80°C (*p* < 0.05). All cases showed a significant decline compared to their initial concentration after 180 days (Figure 4A).

The GAE assay (Figure 4B) showed activity decline after 10 days at +4°C and pH 2.5, while the decline was delayed until day 30 and 60 at −21°C and −80°C, respectively. At pH 6.8, the temperatures of +4°C and −21°C displayed similar decreases starting on Day 10, and at −80°C, no decrease is observed until Day 60 compared to −21°C. The ABTS assay (Figure 4C) revealed no significant change at pH 2.5 and −80°C over 180 days. Nevertheless, slight decreases were observed at 60 days for −21°C and at 20 days for +4°C, both at pH 2.5 (Figure 4B). At pH 6.8, lower antioxidant activity was registered after 30 days at +4°C, correlating with lower gadusol concentration (Figure 4A). Furthermore, there were no significant differences between storage temperatures of −80°C and −21°C after 180 days (Figure 4C).

**FIGURE 5:**
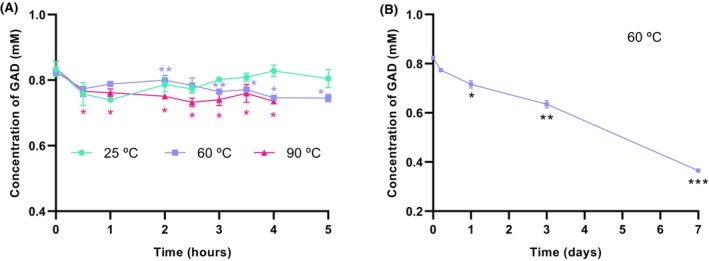
Time profiles of gadusol concentration (mM) under two experimental conditions, (A) heating at three temperatures (+25°C, +60°C, and +90°C) at pH 6.8. (B) Longer heating time at +60°C at pH 6.8. Different asterisks indicate statistical differences between groups (ANOVA with post hoc Tukey test, *p* < 0.05).

Figure 5A shows that over a 5‐h heating experiment, the level of gadusol remained relatively constant at all three temperatures (25°C, 60°C, and 90°C). However, an immediate 10% drop in concentration occurred during the first 30 min. Subsequently, the concentration remained stable at 25°C, whereas a slightly greater decrease was observed at 60°C and 90°C. Overall, the differences in concentration were minor between temperatures. Figure 5B presents the 7‐day experiment at 60°C and pH 6.8, showing that gadusol degradation reached 23% after 3 days and 55% after 7 days.

## DISCUSSION

This study offers valuable insights into the photochemoprotective and antioxidant effects of natural crude extracts from yellowtail amberjack fish (*Seriola lalandi*) roes. Our results identified this aquaculture by‐product as a source of gadusol species, a group of secondary metabolites with photoprotective properties. Additionally, the gadusol yield in the extracts was consistent with previously reported values for other fish species, which ranged between 2.45 and 1.75 mg g^−1^ of dry tissue,^15^ falling within the same order of magnitude. UV–visible absorption analysis allowed the identification of gadusol from its characteristic solvatochromic shift with pH (Figure 1A). This behavior has been interpreted in terms of the displacement of the acid–base equilibrium that relates to both enol‐enolate species (gadusol‐gadusolate), in consistency with the pKa value of 4.25 for gadusol.^35^ HPLC analysis of the extracts confirmed the peak was assigned to gadusol at a retention time of 2.18 min, contrasted with the result for a self‐prepared standard (Figure 1B).

The robust in vitro antioxidant activity of gadusol species in the extracts was confirmed, showing levels comparable to those of reference antioxidants such as trolox and gallic acid, as previously described in similar studies.^10^, ^14^ Assessment of antioxidant activity is a challenging task because of the diverse range of available assays and reaction mechanisms involved.^28^ In the present work, we carried out two electron transfer reactions (ET) (ABTS and Folin–Ciocalteu) and an assay to evaluate the metal‐chelating capacity of the extracts. For the ABTS assay, extended reaction times indicated that the interaction between gadusol and the ABTS radical cation is kinetically less favorable than that of the standard compound Trolox (Table 1), which achieves its maximum activity in a shorter time.^36^ However, the TEAC value at 60 min is similar to that of ascorbic acid.^28^

The Folin–Ciocalteu assay indirectly assesses the antioxidant capacity of a sample by quantifying its total phenolic content, highlighting the role of phenolic groups as electron donors in redox reactions.^25^ The GAE value obtained from this test demonstrated comparable activity to that of the standard of 1 mmol L^−1^ gallic acid (Table 1). In contrast, the GAE value of the ferrous ion chelation assay revealed lower antioxidant activity, suggesting that metal chelation is not the primary mechanism of antioxidant action for gadusol (Table 1).

Given the limited number of in vivo studies reported for gadusol, conducting such assays is crucial to gaining valuable insights into its biological role as a photochemoprotective agent and potential applications. The worm *C. elegans* is widely employed as a model organism in biological research because of its short lifecycle, ease of cultivation, and significant genetic similarity to humans (60%–80%), making it an adaptable and effective system for experimental investigations.^29^, ^37^ The results obtained from these experiments provided key findings into the in vivo effects of gadusol in this model. The gadusol concentration tested in this study was chosen based on levels naturally present in fish roes, which are exposed to UV light, while also considering previous reports on ascorbic acid, a structurally related antioxidant.^38^, ^39^ To contextualize, the two UVB irradiation levels selected in the experiment corresponded to approximately 3 and 15 min of midday summer sun exposure at the latitude of Buenos Aires city,^40^ representing short‐term solar radiation exposure for an individual. This analysis indicated differences in ROS production between animals exposed to higher UVB doses and those treated with 1 mM gadusol (Figure 2A). It has been hypothesized that increased ROS production at supraphysiological levels promotes disease, a condition defined by Sies et al.^41^ as oxidative distress. Massive ROS accumulation triggers oxidative distress, contributing to the development of cellular DNA damage and ultimately leading to cell death. Our results also demonstrated an effect on the mortality test at higher UVB doses, a 10% reduction in mortality (Figure 2B), indicating the potential protective role from gadusol‐containing extracts.

This potential protective effect could be attributed to the presence of this molecule in the extract and its dual mechanism: photoprotection during irradiation and antioxidant activity in the subsequent 24 h of UVB exposure. According to previous studies, gadusol not only intensively absorbs UVR but also exhibits high photostability by rapidly dissipating the photon energy as heat in a non‐radiative relaxation process.^10^ In addition, the ability of gadusol to reduce photoexcited species generated under light exposure could also contribute to its antioxidant capacity. Recently, Orallo et al.^13^ reported the quenching of singlet oxygen by gadusol in water, demonstrating the dominant chemical nature of this process, which is kinetically comparable to that of natural antioxidants such as ascorbic acid. In turn, its photooxidation quantum yield value suggests that gadusolate, the biologically relevant gadusol species, is more efficient as a quencher and acts as a sacrificial antioxidant.^10^

On the other hand, similar molecules such as ascorbate or polyphenols have been shown to reverse oxidative stress induced by metal nanoparticles or hydrogen peroxide in C. *elegans*.^39^, ^42^, ^43^, ^44^ This protective ability could be linked to the shared enolate structure of gadusolate and ascorbate, which allows them to disrupt the peroxyl radical chain reactions. To illustrate gadusol's photoprotective efficacy, we compared our findings to a reported UVB protectant formulation containing tartary buckwheat flavone (TBF), which required an 18‐fold higher dose than the concentration tested in our study to achieve comparable protection against UVB damage in nematodes.^45^ This suggests that gadusol may exhibit relatively stronger activity. However, Rice et al.^18^ evaluated the ability of gadusol as an endogenous antioxidant in zebrafish embryos under oxidative stress induced by hydrogen peroxide, but concluded that the in vivo antioxidant efficacy was not significant.

Our results on the lipofuscin content indicated no protective effect against age‐related damage (Figure 2C). This pigment, commonly referred to as the “age pigment”, is produced naturally throughout the lifespan of an organism. As aging progresses, it aggregates within post‐mitotic cells, resulting in age‐dependent degeneration of various cellular systems. Its accumulation appears to be associated with the inability of cellular proteolytic mechanisms to degrade it during aging.^46^ It is possible that, as the L1 larvae stage was used for the experiment, the concentrations of lipofuscin developed in the organisms were not sufficient to show significant differences over the exposure.

While *C. elegans* provides a holistic organismal model, with high conservation of many human genes and biological mechanisms to assess systemic antioxidant efficacy,^29^ HaCaT keratinocytes serve as a reliable model for studying drug effects and UV responses in human epidermis.^47^ To explore the dose‐dependent implications of gadusol‐rich extracts on UVB‐induced cellular damage, two concentrations of gadusol were initially tested,^48^, ^49^ along with two doses of UVB radiation (Figure S1). The highest UVB dose had a significant impact on the cells; therefore, we chose to proceed exclusively with the lower dose (Figure 3). We found that gadusol (0.01 mM) significantly protected against cell death at a UVB dose of 0.06 J cm^−2^. However, higher concentrations significantly reduced cell viability, with the effect becoming more pronounced at the highest tested dose (Figure 3 and Figure S1). This concentration‐dependent response suggests a complex interaction between gadusol and cellular processes, highlighting the need for further investigation of its mechanisms of action. Yang et al.^50^ reported a similar behavior, observing a cytotoxic effect at the highest concentration of extracts from laver, a red alga (*Porphyra* sp.), which contains MAAs. They attributed this effect to other water‐soluble components present in their extract. Although our extracts underwent substantial purification, the presence of other compounds cannot be entirely ruled out.

To further investigate whether gadusol plays a role in ROS generation after exposure to UVB, we treated HaCaT cells with or without the extract and examined the changes 24 h after UVB irradiation (Figure 3). These results revealed a cytotoxic effect at 0.05 mM gadusol, with no greater protection than at 0.01 mM. Regarding the question investigated whether gadusol acted mainly as a photoprotector or antioxidant, the results suggest that gadusol, at a concentration of 0.01 mM, may partially protect cells against damage after irradiation, as an antioxidant, in line with previous reports.^13^ In addition, a significant difference in cell viability was observed when a 0.05 mM concentration of gadusol was present within 24 h after irradiation.

Given the differences observed in the post‐irradiation treatments in the cellular experiments, we wondered which metabolic mechanism might underlie this response. There is background information on certain metabolic mechanisms of MAAs that are biosynthetically and structurally related to gadusol. As previously mentioned, extracts from laver containing palythene, porphyra‐334, or shinorine showed similar effects, lowering apoptosis after UVB irradiation.^48^, ^51^ All of these MAAs are imine‐type disubstituted with imino groups,^52^ a difference between gadusolate, which only bears an enolate‐cyclohexane structure. According to these reports, the MAAs promote the enhanced transcriptional regulation of Nrf2‐targeted genes after exposure to UVR‐induced oxidative stress. This indicates their role as direct antagonists of Keap1‐Nrf2 binding, providing cytoprotective functions by activating the Keap1‐Nrf2‐ARE pathway.^53^, ^54^ In particular, the antioxidant properties of the MAA shinorine help alleviate intracellular ROS levels by activating the Nrf2 pathway.^19^ The same authors studied the binding of DGR domains of Keap1 in zebrafish proteins by molecular docking, with shinorine and porphyra‐334 demonstrating their Keap1‐Nrf2 disrupting interaction.

We conducted a molecular docking study to evaluate whether gadusolate can bind to the Nrf2‐binding domain of Keap1, potentially inhibiting the Keap1–Nrf2 interaction. Our docking results showed different amino acid residue interactions compared to the interaction between shinorine and Keap1.^19^ Although gadusolate may form more hydrogen bond interactions with Keap1, it is not in the DGR domain like those of shinorine, and its binding may be less effective or insufficient. Moreover, the final minimized structure of gadusolate, optimized in the presence of the protein, differs from that reported by Losantos et al^14^ Further investigations are required to better understand the potential mechanisms of action.

From the point of view of the potential applications of the gadusol‐containing extracts reported here, a deeper understanding of their stability under different temperatures and storage conditions is essential. Stability studies could provide critical insights into the optimal storage methods, helping to preserve their photoprotective and antioxidant properties. According to our results, the stability was generally greater at −80°C, with no differences between the two pH analyzed, followed by −21°C, with almost the same stability over the first 60 days (Figure 4). The set of −4°C and pH 6.8 describes the least favorable conditions for the storage of extracts. Notably, analysis at pH 6.8 and +4°C was stopped after 30 days due to fungal growth contamination, highlighting the influence of this condition and the carbon and nitrogen content of the medium that allows fungal development.

To further explore the degradation process, an accelerated experiment under higher temperatures was performed (Figure 5). This evaluation showed the remarkable stability of gadusol in crude extract solutions, even when subjected to extended periods of elevated temperatures. These results suggest that gadusol may be well‐suited for applications requiring long shelf life and stability, enhancing its potential as a reliable active ingredient in formulations exposed to variable environmental conditions.

## CONCLUSION

In conclusion, this study provides relevant findings on gadusol‐rich extracts derived from a natural marine source and evaluated multiple criteria to propose them as viable alternatives to synthetic sunscreens. The results of our in vitro experiments are in line with previously reported robust antioxidant activity of gadusol. Effective UVB photochemoprotective activity was confirmed, with a 10% reduction in mortality observed at the highest UVB dose in *C. elegans*, besides protection exceeding 50% in human keratinocytes being verified at a gadusol concentration of 0.01 mM. Stability studies are crucial for ensuring product efficacy and safety, as our findings confirm that gadusol exhibits general good stability over 180 days, thus supporting the promising development of gadusol‐based formulations. These findings enhance our understanding of gadusol in crude extracts while reinforcing their potential benefits for applications in cosmetics, pharmaceuticals, and/or nutraceuticals, providing valuable insights into a scarcely explored area of research.

## DISCLOSURE

During the preparation of this work, the author(s) used Paper Pal (www.paperpal.com) to revise the English. After using this tool/service, the author(s) reviewed and edited the content as needed and take(s) full responsibility for the content of the publication.

## Supporting information


Figure S1.

## Data Availability

The data that support the findings of this study are available from the corresponding author upon reasonable request.
