# Supplementary material for: Exploring natural sunscreens: UVB protection and antioxidant properties in gadusol‐rich fish roes extracts
Source: Photochem Photobiol. 2025 Oct 6;102(4):877–88. doi: 10.1111/php.70042 (PMC13383027; doi:10.1111/php.70042)
Supplement: Supplementary file 1 — Figure S1. [file PHP-102-877-s001.docx]

**Supplementary material**

**Exploring natural sunscreens: UVB protection and antioxidant properties in** **gadusol-rich fish roes extracts**

**Rocío Isla Naveira^a,b,*^, Gabriela Hollmann^c^, José María Monserrat^c,d^ , Ana Paula S. Votto ^c,d^, Julie Madeiros da Silveira^c,d^, Andressa Mai Matsumoto^c,d^, Lais Zortéa^c,d^, Andy Joel Taipe Huisa^c,d^, Agueda E. Massa^a,b^, M. Sandra Churio^e,f^**

^a^ Instituto Nacional de Investigación y Desarrollo Pesquero (INIDEP) Paseo Victoria Ocampo Nº 1, B7602HSA, Mar del Plata, Argentina.

^b^ Instituto de Investigaciones Marinas y Costeras (IIMyC), UNMDP – CONICET, Rodríguez Peña 4046, B7602GSD, Mar del Plata, Argentina

^c^ Instituto de Ciências Biológicas (ICB), Universidade Federal do Rio Grande—FURG, Av. Itália km 8 s/n, Cx.P. 474, Rio Grande CEP 96200-970, RS, Brasil

^d^ Programa de Pós-graduação em Ciências Fisiológicas, Universidade Federal do Rio Grande—FURG, RS, Brasil.

^e^ Departamento de Química y Bioquímica, FCEyN, UNMDP, Deán Funes 3350, B7602AYL, Mar del Plata, Argentina

^f^ Instituto de Investigaciones Físicas de Mar del Plata (IFIMAR), UNMDP – CONICET, Deán Funes 3350, B7602AYL, Mar del Plata, Argentina

*Corresponding author:

Instituto de Ciências Biológicas (ICB), Universidade Federal do Rio Grande—FURG, Av. Itália km 8 s/n, Cx.P. 474, Rio Grande CEP 96200-970, RS, Brasil.

Email: [josemmonserrat@gmail.com](mailto:josemmonserrat@gmail.com)

**List of Figures**

**Fig S1.** Viability (%) of HaCaT cells after 24 h of irradiation with two different doses of UVB and two gadusol concentrations.

**Fig S2.** Molecular docking analysis showing the interaction between Keap1 and gadusolate. (A) 3D representation of the interbond distance between gadusolate and amino acid residues. (B) 2D representation of the interaction between gadusolate and Keap 1a and 1b.

**
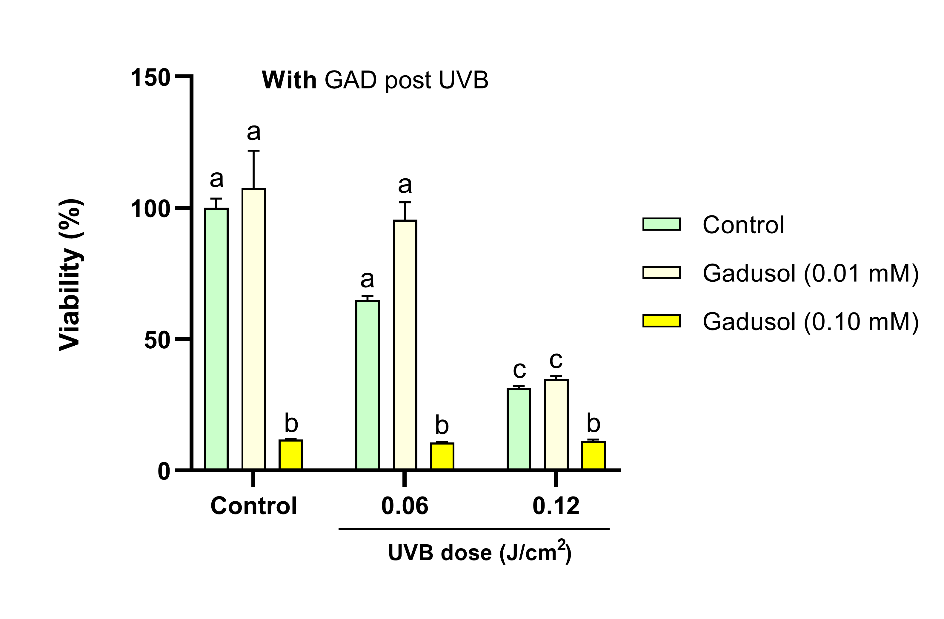
**

**Fig S1.** Viability (%) of HaCaT cells after 24 h of irradiation with two different doses of UVB and two gadusol concentrations.


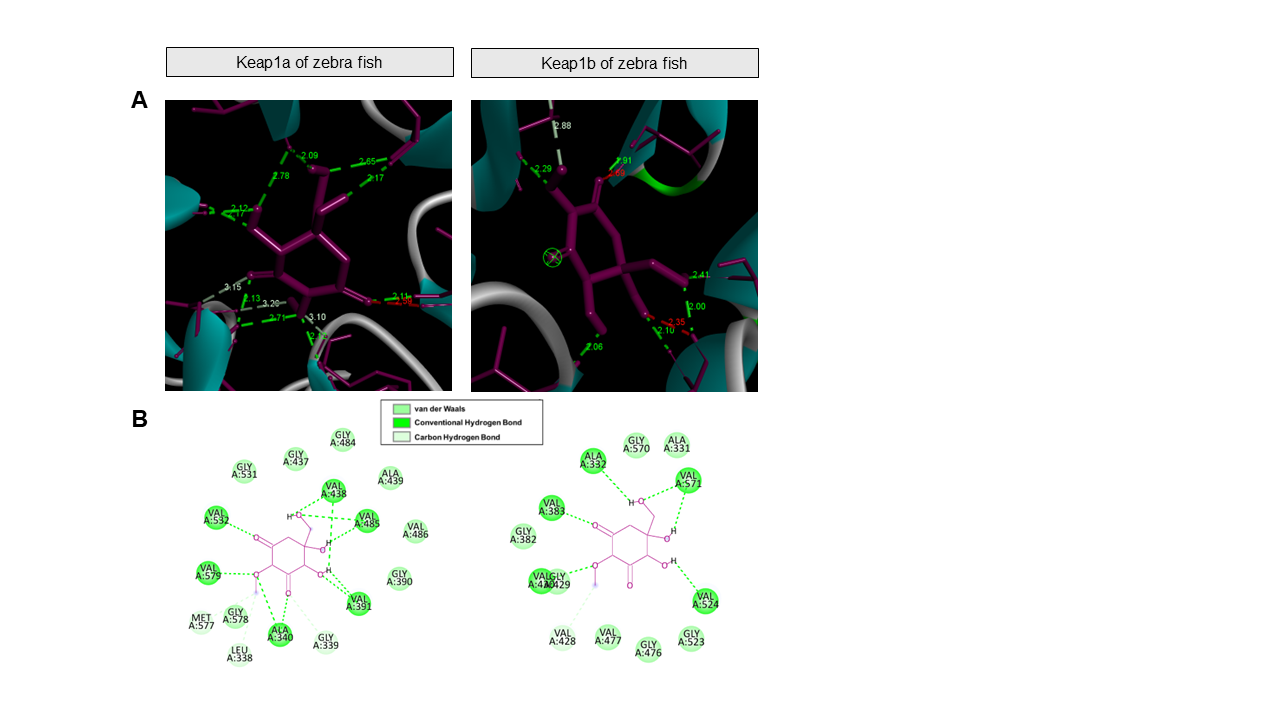


**Fig S2.** Molecular docking analysis showing the interaction between Keap1 and gadusolate. (A) 3D representation of the interbond distance between gadusolate and amino acid residues. (B) 2D representation of the interaction between gadusolate and Keap 1a and 1b.
